# Clinical and Radiographic Evaluation of Brånemark Implants with an Anodized Surface following Seven-to-Eight Years of Functional Loading

**DOI:** 10.1155/2013/583567

**Published:** 2013-03-07

**Authors:** David Gelb, Bradley McAllister, Pirkka Nummikoski, Massimo Del Fabbro

**Affiliations:** ^1^West Hartford Hospital and Private Practice, West Hartford, CT 06119, USA; ^2^Department of Periodontology, Oregon Health Sciences University and Private Practice, Portland, OR 97224, USA; ^3^The University of Texas Health Science Center, San Antonio, TX 78229-3901, USA; ^4^Department of Biomedical, Surgical and Dental Sciences, Research Centre for Oral Health, IRCCS Istituto Ortopedico Galeazzi, Università degli Studi di Milano, 20161 Milano, Italy

## Abstract

The aim of this study was to evaluate the clinical and radiographic long-term outcomes of dental implants with an anodized TiUnite surface, placed in routine clinical practice. Two clinical centers participated in the study. One hundred and seven implants (80 in the maxilla and 27 in the mandible) in 52 patients were followed in the long term. Both one- and two-stage techniques were used for 38 and 69 implants, respectively. Thirty-eight single tooth restorations and 22 fixed partial prostheses were delivered, according to a delayed loading protocol, within 4 to 12 months since implant placement. All implants were stable at insertion and at the long-term follow-up visit, which occurred between 7 and 8 years of functional loading. The mean followup was 7.33 ± 0.47 years. The mean marginal bone level change at the long-term followup as compared to baseline was 1.49 ± 1.03 mm. No implant failure occurred. Healthy peri-implant mucosa was found around 95% of implants, whereas 91% of implants showed no visible plaque at the implant surfaces at the long-term followup. The study showed that dental implants with the TiUnite anodized surface demonstrate excellent long-term clinical and radiographic outcomes.

## 1. Introduction

The long-term success of the original Brånemark machined-surfaced osseointegrated dental implants is clearly demonstrated in the scientific literature. Numerous clinical evidences prove the consistency of the guidelines suggested in the original Brånemark protocol, where osseointegration of dental implants can be achieved and maintained for a long time under functional loading [1–6]. Over the years, the original Brånemark protocol underwent many modifications that further increased the applicability and predictability of implant treatment. For example, the reduction of the healing period with the advent of early and immediate loading protocols, and the placement of implants in fresh postextraction sockets, or in regenerated bone, allow clinicians to extend implant therapy to a broader population of patients as well as improve the clinical success of such treatment. The macroscopic and microscopic features of the fixtures have also dramatically changed, due to a series of modifications aimed at optimizing the mechanical anchorage as well as the osseointegration process in different clinical situations. The role of implant surfaces has long been considered as critical for the success of the treatment, which relies upon a proper osseointegration [7, 8]. It has been demonstrated that titanium per se does not establish an intimate direct contact with the surrounding bone [9, 10]. Conversely, the surface layer of titanium oxide, which spontaneously forms when the surface is exposed to the atmosphere, is highly biocompatible and permits implant osseointegration [11]. One of the most significant breakthroughs in implant dentistry was the introduction of implants having a textured surface. The latter were developed with the aim of allowing for more predictable and possibly faster osseointegration. Many histological studies demonstrated that implants with rough surface develop a higher bone-to-implant contact in the early healing phase, have similar marginal bone remodelling during their first year of function, and may achieve better survival rates in demanding situations when compared to implants with machined surfaces. [12–15] Furthermore, a higher torque is necessary to remove rough-surfaced implants in comparison to machined ones [16–20].

For these reasons, an implant with a highly crystalline, phosphate-enriched titanium oxide layer, characterized by a microstructured surface with 1–10 *µ*m pores, such as the TiUnite (Nobel Biocare Göteborg, Sweden), is expected to be particularly suitable for obtaining a successful and long-lasting osseointegration [21, 22]. Such an anodized surface, which was launched in the year 2000, has been proven to increase initial bone-to-implant contact and has shown osteoconductive properties resulting in improved maintenance of implant stability in the healing phase when compared to machined titanium implants [21–24]. 

Ivanoff et al. [25], in a study evaluating the response of human jawbone to microimplants with turned or oxidized surface installed in maxillae and mandibles, found that bone-to-implant contact was higher for implants with TiUnite surface when compared to machined Brånemark implants. The former also demonstrated significantly more bone within the threaded area with respect to machined micro-implants. In another study on mini-pigs, Zechner et al. [21] compared titanium implants with three different surfaces: anodized, hydroxyapatite (HA) coated and machined. The authors found that both the anodized surfaced and the HA-coated implants showed significantly higher bone-to-implant contact when compared to the machined-surfaced implants [21].

Although moderately rough-surface implants are widely utilized nowadays and clinically documented in a number of scientific publications, some controversial issues concerning possible undesired effects still exist. A main concern is the impact of surface roughness on the peri-implant tissues health. In fact, although peri-implantitis is one of the prevalent biological complications occurring around machined-surfaced implants [26], some studies suggest that there may be an increased risk of peri-implantitis with rough-surfaced implants due to bacterial adhesion which increases the risk of biofilm accumulation onto the implant surface. In experimental studies adopting a ligature-induced peri-implant disease dog model, it was observed that different types of rough-surfaced implants display a higher bone loss than machined-surfaced implants, once the ligature is removed [27, 28]. 

In a clinical study, Åstrand et al. [29] found that implants with a TPS surface (titanium plasma sprayed) were more susceptible to peri-implantitis than machined-surfaced implants after three years of function. On the other hand, a 5-year-long followup study done by Wennström et al. [30] showed no increase in peri-implantitis for a rough-surfaced implant (TiO blasted) when compared to machine-surface implants. Another study by Vroom et al. [20] compared TiO-blasted rough surface implants to machine-surface implants reporting soft and hard tissue responses. After 12 years of followup, they found no significant difference between the two groups. The authors found a small amount of calculus around both types of implants, as well as nearly similar probing depths and no significant vertical bone changes in both implant systems. 

Several short-term clinical reports of anodized implants in different clinical situations and under different loading protocols have been published in the literature showing safety and effectiveness of such implant type after 6 to 36 months of followup. In fact, survival rates of 96% to 100% have been reported for early- or immediately-loaded implants, and similar survival rates have been reported for compromised clinical situations such as limited bone volume, low density bone, and/or concomitant augmentation procedures [31–34]. 

Although a number of publications report long-term outcomes of rough-surfaced implants in the dental literature, little is known about the modifications of tissues surrounding implants with anodized surface after at least five years of function [35–40]. 

The aim of this two-center study was to assess clinical and radiographic outcomes of anodized implants after 7 to 8 years of functional loading. 

## 2. Materials and Methods 

A retrospective analysis of patients who had Brånemark System TiUnite implants placed earlier than 2004 was conducted in 2010 based on the review of clinical charts from two centres in private practice. The practitioners at the two centres were standardized, with similar expertise in implant dentistry, and followed the same clinical protocol. All patients were treated according to the principles embodied in the Helsinki Declaration of 1980. Patients were selected according to the standard inclusion criteria for implant treatment adopted in the clinics: older than 18 years; physically and psychologically able to undergo conventional implant surgery and restorative procedures (American Society of Anaesthesiologists (ASA) class I or II). For the present study edentulous patients were selected partially treated by means of implant-supported rehabilitations, who had Brånemark System TiUnite implants placed earlier than 2004 and peri-apical X-rays taken at implant insertion. Exclusion criteria were presence of uncontrolled systemic disease such as diabetes mellitus or bone metabolic disease; smoking of 20 or more cigarettes a day; head or neck radiotherapy 12 months prior to surgery; heavy parafunctions (e.g., bruxism); inadequate bone volume and quality that would require reconstruction procedures; a past or current administration of intravenous bisphosphonates; poor oral hygiene and motivation. 

Presurgical clinical assessment of the edentulous region included periapical radiographs, panoramic radiographs, and/or computerized tomography. Overall, 113 implants were consecutively placed in 57 patients. The implants were placed from August 2002 to September 2003. Twenty-five patients were treated and followed in center 1, while 32 patients were treated in center 2, but five of them did not return at the followup visit so that only 27 patients could be evaluated. In summary, 107 implants, 80 in the maxilla (74.8%) and 27 in the mandible (25.2%), were evaluated in 52 patients at the 7-8 years followup. Thirty-eight implants (36%) supported single tooth restorations, and 69 implants (64%) supported 22 fixed partial prostheses (Table 1). Seventy-two implants (67.3%) were evaluated clinically and radiographically after seven years and 35 implants (32.7%) after eight years of functional loading. Seventy implants (65.4% of cases) were placed in healed sites and 37 (34.6%) in extraction sites. Thirty-eight implants (35.5% of cases) were placed using a one-stage surgical approach, while 69 implants (64.5%) were placed using a two-stage surgical approach. 

### 2.1. Surgical and Prosthodontic Procedures

Each patient was given a detailed explanation regarding the treatment and signed a written consent form prior to implant placement. Local anesthesia was induced using Xylocaine with 1 : 100,000 Epinephrine or Carbocaine. Immediate placement of implants occurred in fresh postextraction sockets (Type 1 implants according to the classification proposed by Hämmerle in 2004 [41]). The extraction sites were carefully debrided and granulation tissue was entirely removed. Site preparation followed the sequence recommended by the manufacturer. Healing abutments were placed at the time of implant placement in order to guide soft tissue development during the healing phase. 

Prosthetic restorations were delivered according to a delayed loading protocol. The mean time elapsing from implant placement to the prosthetic phase was 4.2 months in the mandible and 6.4 months in the maxilla. After definitive abutment connection, conventional impression techniques were performed, followed by fabrication of the final prosthesis. The longest time from implant surgery to final prosthesis delivery was 12 months. A total of 38 implant-supported single-crown and 22 fixed dental prosthesis were delivered. 

### 2.2. Followup

*Radiographic Evaluation*. Digital periapical radiographs were taken using Rinn detector standardized holders at implant insertions and at 7 to 8 year after surgery. 

Each radiograph was corrected for distortion and image magnification to match the true millimeter dimensions of the alveolar bone by using the known implant thread distances in the image as calibration reference (for Brånemark Mk III and IV the thread distance is 0.6 mm). 

Once images were calibrated, the bone levels were measured on the mesial and distal sides of each implant from the implant shoulder as reference point to the first coronal bone-to-implant contact. The image calibrations and measurements were performed using image analysis software ImageJ version 1.43u [42].

Bone level change was estimated by calculating the difference between baseline and followup values. 

*Peri-Implant Evaluation*. Each implant site was clinically evaluated using the following parameters. The peri-implant plaque index (PI) quantified the plaque present around all abutments at or below the crest of the peri-implant mucosa using a modified Silness and Löe technique [43, 44]. The gingival index (GI) was used to assess the presence or absence of inflammation of the soft tissue around the same four sites of each implant: mid-facial, mid-lingual, mid-mesial and mid-distal [43]. The scores for peri-implant mucosa were: 0 = normal mucosa, 1 = bleeding on superficial probing, and 2 = discoloration, spontaneous bleeding. The peri-implant indexes were recorded at the latest followup, 7 to 8 years after the implant placement. The values recorded at each implant site were averaged so as to have a single mean value for both the PI and GI indexes per each implant. 

*Statistical Evaluation.* The difference, in marginal bone loss between followup data and baseline and between restoration types (partial bridges and single-tooth restorations) at the latest followup were statistically evaluated by means of the Mann-Whitney *U* test. All significance tests were two tailed and conducted at the 5% significance level. All analyses were performed using SPSS (version 19.0; IBM Corporation; Armonk, NY, USA).

## 3. Results 

Of the initial sample of 57 patients and 113 implants, five patients, accounting for six implants, were lost to followup due to personal reasons. Only data of patients that could be evaluated at the long followup have been analyzed and are presented in this paper. The study sample of 52 patients (21 males and 31 females, mean age 64.09 ± 11.40 years, range 35 to 82 years) included 3 smokers, 4 patients with controlled type I diabetes, 4 patients with periodontitis at the time of implant placement, and 41 healthy subjects. The implants inserted consisted of 96 Brånemark System MKIII (90%) and 11 MK IV (10%) TiUnite implants. Implant location in the maxilla and mandible is shown in Table 1.

No implant failure was recorded. All implants were stable at implant insertion and at the longest followup (7-8 years). The mean followup was 7.33 ± 0.47 years.  Table 2 shows the cumulative survival analysis throughout the observation period. No symptoms of peri-implantitis were reported, and peri-apical radiographs demonstrated stability of the peri-implant bone levels. The mean marginal bone loss was 1.49 ± 1.03 mm for 77 implants after 7-8 years. It was not possible to perform any bone level measurement at the latest followup for 30 implants due to either missing radiographs or poor quality of the radiographs. Marginal bone loss was slightly higher around implants supporting partial bridges (1.66 ± 1.00 mm, *n* = 20) as compared to single-tooth restorations (1.42 ± 1.04 mm, *n* = 57). This difference was not statistically significant (*P* > 0.05).

**Figure 1:**
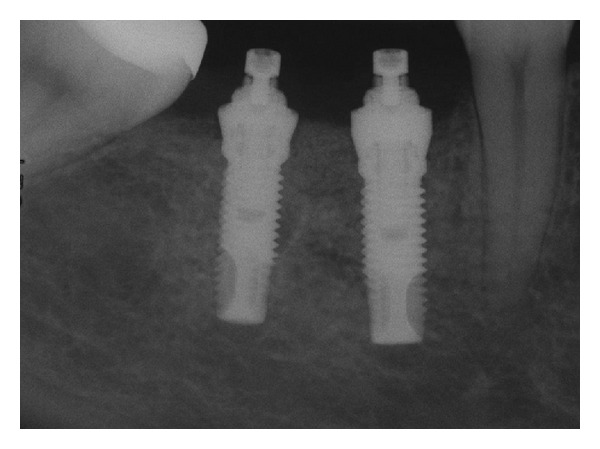
Periapical radiograph at abutment connection.

**Figure 2:**
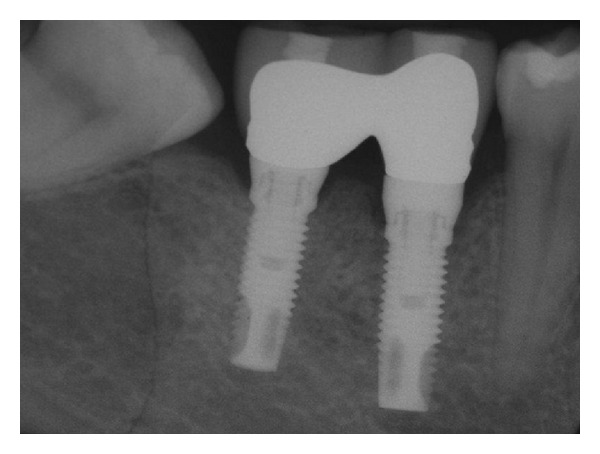
Periapical radiograph of the implants 8 years after implant placement. Substantial stability of peri-implant bone levels can be appreciated.

**Figure 3:**
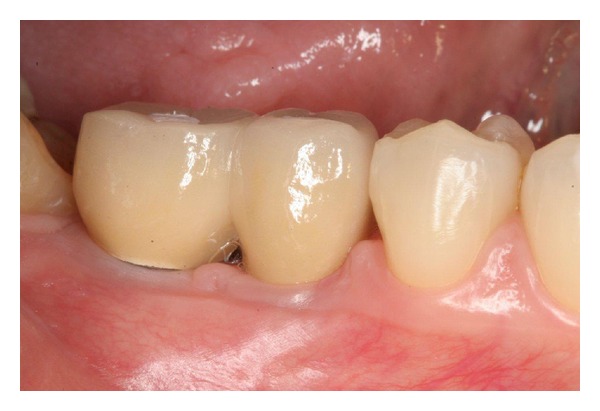
Intraoral view of the implant crowns 8 years after implant placement. Peri-implant soft tissues appear healthy.

A healthy, noninflamed peri-implant mucosa was observed at 102 implants (95.3% of cases), while only 5 (4.7%) showed bleeding on probing. Ninety-seven implants (90.7% of cases) showed absence of visible plaque which was detected at 10 implants (9.3%) at 7-8 year followup (Table 3). No prosthetic complication nor severe adverse events were reported throughout the long-term follow-up visit. Figures 1 to 3 illustrate a clinical case evaluated after 8 years of function, showing stable peri-implant bone levels (Figures 1 and 2) and healthy peri-implant soft tissues (Figure 3).

## 4. Discussion

Scarce documentation exists on the long-term success of anodized implants in the literature, although many short-term clinical reports show favorable results. The present retrospective study, even in face of a limited sample size, demonstrated that implants with oxidized surface supporting single crowns and fixed partial reconstructions may achieve and maintain an excellent clinical performance up to 8 years of function. 

The conventional implant success criteria proposed by Albrektsson et al. in 1986 require that implants display immobility, absence of peri-implant radiolucency, and marginal bone loss not exceeding 1.5 mm after the first year of loading and up to 0.2 mm yearly [45]. Furthermore, an implant system can be considered successful if 85% of the implants meet these criteria under loading for a period of at least 5 years [45]. Buser et al. in 1991 proposed similar implant success criteria, without specifying the tolerated marginal bone loss over time: absence of disturbances, pain, foreign body sensation, or altered sensitivity, absence of recurrent peri-implant infection in association with suppuration, absence of mobility, and absence of continuous radiotransparency along the implant profile [46]. In the present study, 94.8% of the implants radiographically evaluated met the success criteria established by Albrektsson et al. [45], since four implants out of 77 displayed a marginal bone loss exceeding 3 mm after at least seven years of placement. The present longitudinal data, despite the limited sample size, may contribute to substantiate the successful performance of the TiUnite surface in the long term. 

The results of this study are in accordance with the 5-year data reported by other authors who evaluated the clinical outcomes of implants with the TiUnite Surface. Calandriello and Tomatis [47] evaluated immediately placed anodized implants in 33 patients and found a mean marginal bone loss of 1.17 mm after 5 years. In another retrospective study, Friberg and Jemt [35] compared 280 TiUnite implants to 110 machined implants in 111 patients and found marginal bone loss after 5 years to be 0.75 and 0.6 mm, respectively, with no significant difference between the two types of implants. Moreover, the prevalence of implants with bony defects greater than 2 mm was very low for both types of implants [35]. The long-term data of bone loss found in the present study is also in accordance with those reported by several other studies which evaluated various rough-surfaced implants [36, 48–51]. A study done by Rasmusson et al. reviewed TiO-blasted roughened implants after 10 years and found no increase in progressive bone loss or peri-implantitis [51]. Five- and seven-year results of a study on 38 patients have been published, demonstrating good long-term outcome for TiUnite implants under immediate loading protocol [36, 37]. Fifty-one fixed prostheses (30 partial dentures, 20 single crowns, and one mandibular complete denture) supported by 102 implants placed mainly in posterior regions of the jaws and primarily in soft bone quality were loaded the same day of intervention. GBR was performed in 66 sites (64.7%) with exposed implant threads. Three maxillary implants were removed in one patient after 8 weeks due to infection close to a GBR site, resulting in 97.1% overall cumulative implant survival rate. Few complications were reported over time. The mean marginal bone loss was 1.51 ± 1.00 mm after 7 years. Such value is very similar to that reported in the present study, but the loading temporization was shorter. In spite of the differences in protocol, the occurrence of three early failures and a greater proportion of complications, the clinical results of the study by Glauser et al. [36, 37] are comparable to the current study, confirming the predictability of the TiUnite surface in the long term. The latter is further confirmed by two recent articles reporting excellent results of studies on implants with TiUnite surface after 10 years of function [39, 40]. The study by Degidi et al. [48] reported 97.6% of implant survival on 210 immediately loaded implants placed in 59 patients, while Östman et al. [49] found 99.2% implant survival on 121 implants (of which 20% immediately loaded) in 46 patients after 10 years.

In the current study, the large majority of implants (95.3%) had a normal peri-implant mucosa, and 5 implants (4.7%) had bleeding on superficial probing of the peri-implant mucosa. The long-term data in the present study suggests that the TiUnite surface over time behaves comparably to (or better than) machined-surface implants. These results are comparable to other long-term studies that looked at peri-implant tissues around rough-surfaced versus machined implants. Vroom et al. [20] investigated long-term differences between TiO-blasted and machined implants in regards to peri-implant health in the same patient. After 12 years, the rough-surfaced and machined-surface implants showed no significant differences in marginal bone loss and soft tissue health, including plaque, calculus, bleeding, and probing pocket depth. Another study was done by Åstrand et al. [29], found that the rough-surfaced TPS implants were more susceptible to peri-implantitis when compared to machined-surfaced implants in the same patient after 3 years. The results of the current study do not support the hypothesis that rough-surfaced implants may cause more peri-implantitis than machined implants. Another study done by Karoussis et al. [52], compared biological complications of three different ITI implants. Over a 10-year period, the three implants showed an incidence of peri-implantitis ranging from 10% to 29% when including strict radiographic and clinical parameters [52]. These values were slightly worse than the values found in the current study analyzing anodized implants. 

The proposed danger of rough-surfaced implants is their potential to accumulate more plaque than smooth-surfaced implants. Few studies have found that in general rough-surfaced implants promote more plaque accumulation and subsequent bone loss than machined-surfaced implants. Quirynen et al. [53] performed a literature review which focused on implant surface roughness as related to peri-implantitis. They stated that rough-surfaced implants were more susceptible to late implant loss and/or marginal bone loss in patients with a history of periodontitis than were minimally rough implants. 

Despite the early general concerns of higher plaque accumulation on rough-surfaced implants in comparison to machined implants, the current study showed low levels of plaque and marginal bone loss around anodized implants. The stable peri-implant mucosal health found in this study may be attributed to such low plaque levels found in the majority of the anodized implants in this patient population. 

## 5. Conclusion

The clinical and radiographic outcomes of the present study showed high implant success rates and healthy peri-implant mucosa, confirming the long-term predictability of implants with moderately rough surface.

## Figures and Tables

**Table 1:** Distribution of the implants used in this study.

Implant type	Implant length, mm	Maxilla, number of implants	Mandible, number of implants
Brånemark System Mk IV TiUnite 4.0 mm	7	2	0
	10	7	0
	11.5	1	0
	13	1	0

Brånemark System Mk III TiUnite 4.0 mm	8.5	2	1
	10	21	11
	11.5	1	3
	13	28	10
	15	13	2
	17	1	0
	18	2	0

Brånemark System MkIII TiUnite 3.75 mm	15	1	0

Total		80	27

**Table 2:** Kaplan-Meier (life-table) analysis.

Time interval	Followed implants	Failed implants	Implants lost to followup	Cumulative survival rate
Insertion–6 months	113	0	0	100%
6 months–1 year	113	0	0	100%
1-2 years	113	0	4	100%
2-3 years	109	0	0	100%
3-4 years	109	0	0	100%
4-5 years	109	0	0	100%
5-6 years	109	0	2	100%
6-7 years	107	0	0	100%
7-8 years	107	0	0	100%

**Table 3:** Soft tissue scores after 7-8 years.

	Number of implants	%
Gingival index		
0 = normal mucosa	102	95
1 = bleeding on superficial probing	5	5
2 = discoloration, spontaneous bleeding	0	0
Plaque index		
Yes	10	9
No	97	91
